# Subventricular zones: new key targets for glioblastoma treatment

**DOI:** 10.1186/s13014-017-0791-2

**Published:** 2017-04-20

**Authors:** J. Khalifa, F. Tensaouti, A. Lusque, B. Plas, J.-A. Lotterie, A. Benouaich-Amiel, E. Uro-Coste, V. Lubrano, E. Cohen-Jonathan Moyal

**Affiliations:** 1 Department of Radiation Oncology, Institut Universitaire du Cancer de Toulouse – Oncopôle/Institut Claudius Regaud, 1 avenue Irène Joliot-Curie, Toulouse Cedex, 31059 France; 2 Toulouse NeuroImaging Center, ToNIC, Université de Toulouse, INSERM, Université Paul Sabatier, Toulouse, France; 3 Department of Biostatistics, Institut Universitaire du Cancer de Toulouse – Oncopôle/Institut Claudius Regaud, 1 avenue Irène Joliot-Curie, Toulouse Cedex, 31059 France; 4 Department of Neurosurgery, Institut Universitaire du Cancer de Toulouse - Purpan, Place du Docteur Baylac, Toulouse Cedex, 31059 France; 5 Department of Nuclear Medicine, CHU Rangueil, 1 avenue du Pr Jean Poulhès TSA 50032, Toulouse Cedex, 31059 France; 6 Department of Medical Oncology, Institut Universitaire du Cancer de Toulouse – Oncopôle/Institut Claudius Regaud, 1 avenue Irène Joliot-Curie, Toulouse Cedex, 31059 France; 7 Department of Pathology, Institut Universitaire du Cancer de Toulouse – Oncopôle/Institut Claudius Regaud, 1 avenue Irène Joliot-Curie, Toulouse Cedex, 31059 France; 8 Université Paul Sabatier, Toulouse III, 118 route de Narbonne, Toulouse, 31062 France; 9 INSERM U1037, Centre de Recherche contre le Cancer de Toulouse, 1 avenue Irène Joliot-Curie, Toulouse Cedex, 31059 France

**Keywords:** Glioblastoma, Radiotherapy, Stem-cell niche, Subventricular Zone, Prognostic factors

## Abstract

**Background:**

We aimed to identify subventricular zone (SVZ)-related prognostic factors of survival and patterns of recurrence among patients with glioblastoma.

**Methods:**

Forty-three patients with primary diagnosed glioblastoma treated in our Cancer Center between 2006 and 2010 were identified. All patients received surgical resection, followed by temozolomide-based chemoradiation. Ipsilateral (iSVZ), contralateral (cSVZ) and bilateral (bSVZ) SVZs were retrospectively segmented and radiation dose-volume histograms were generated. Multivariate analysis using the Cox proportional hazards model was assessed to examine the relationship between prognostic factors and time to progression (TTP) or overall survival (OS).

**Results:**

Median age was 59 years (range: 25–85). Median follow-up, OS and TTP were 22.7 months (range 7.5–69.7 months), 22.7 months (95% CI 14.5–26.2 months) and 6.4 months (95% CI 4.4–9.3 months), respectively. On univariate analysis, initial contact to SVZ was a poor prognostic factor for OS (18.7 vs 41.7 months, *p* = 0.014) and TTP (4.6 vs 12.9 months, *p* = 0.002). Patients whose bSVZ volume receiving at least 20 Gy (V20Gy) was greater than 84% had a significantly improved TTP (17.7 months vs 5.2 months, *p* = 0.017). This radiation dose coverage was compatible with an hippocampal sparing. On multivariate analysis, initial contact to SVZ and V20 Gy to bSVZ lesser than 84% remained poor prognostic factors for TTP (HR = 3.07, *p* = 0.012 and HR = 2.67, *p* = 0.047, respectively).

**Conclusion:**

Our results suggest that contact to SVZ, as well as insufficient bSVZ radiation dose coverage (V20Gy <84%), might be independent poor prognostic factors for TTP. Therefore, targeting SVZ could be of crucial interest for optimizing glioblastoma treatment.

## Background

Glioblastoma is the most common primary brain tumor among adults, with poor outcome following chemoradiotherapy, despite deeper insights into molecular biology and significant advances in therapeutics [1]. Like other cancers, glioblastomas are characterized by a high intratumoral heterogeneity in a wide range of phenotypic and functional features [2–4]. The stochastic model has traditionally been accepted as the basis of tumor heterogeneity, wherein a small population of genetically unstable clonal cells randomly acquires the appropriate somatic mutations necessary to confer extensive proliferative capabilities [5]. However, recent evidence suggests that according to the alternative cancer stem cell model [6], glioblastomas are rather organized hierarchically as they contain a subpopulation of cancer cells with stem cell characteristics, including self-renewal capacity and multilineage potency, as well as tumor-initiation/proliferation and migration ability [7–11].

The origin of brain tumor stem cells is still controversial, but neural stem cells are highly likely to be candidate as both populations share many similarities, including molecular pathways (Notch receptor activation [12, 13], inactivation of PTEN tumor suppression gene [14]) and markers of gene expression (like Nestin, CD133 or Sox [15]). A growing body of evidence has also suggested that any glioma cell could acquire a stem cell phenotype in response to hypoxia, or even radiation, contributing to GBM radioresistance [16–18]. Stem cells of various tissues exist within protective niches composed of a number of differentiated cell types providing direct cell contacts and secreting factors that maintain stem cells primarily in a quiescent state [19]. It is then hypothesized that tumor stem cells might arise from normal stem cells that have acquired mutations which enable them to escape from niche control or after deregulation of extrinsic factors within the niche, leading to uncontrolled proliferation of stem cells and tumorigenesis [20–22]. The subventricular zone (SVZ) is the largest niche of neurogenesis in the adult mammalian brain [23, 24], so that an emerging hypothesis is that brain tumor stem cells could derive from the SVZ [11]. It should be noted that the subgranular zone abutting the hippocampal dentate gyrus is a secondary nich in adult mammalian brains, but where neurogenesis only occurs in foci closely associated with blood vessels [25].

Many data have already provided interesting relationships between SVZ involvement in glioblastoma and outcome or pattern of relapse, strongly suggesting that tumors contacting SVZ are associated with multifocal presentation at diagnosis, multifocal or distant progression [26–29] and above all decreased survival [27, 29–31]. This stresses the need to better understand the role of the SVZ as a potential source of glioblastoma through initiation and promotion, a potential source of relapses through repopulation of tumor, and as a target for glioblastoma treatment.

Glioblastoma stem cells are known to be inherently resistant to chemotherapy and radiotherapy [32, 33]. One strategy to overcome glioblastoma stem cells radioresistance could consist in increasing the radiation dose to the SVZ. The impact of incidental radiation dose to the SVZ during the course of a standard chemoradiotherapy on the outcome of patient has already been assessed, but mostly in inhomogeneous series and with somewhat contradictory results [34–41]. These results are all the more confusing that such irradiation to neural stem cells is source of radiation-induced neurotoxicity [42], and is in contradiction with hippocampal sparing strategies [43].

Therefore, the objective of this retrospective study was to identify SVZ-related prognostic factors of survival and patterns of recurrence, with regard to both surgical and radiotherapeutic management, to better integrate SVZ in the global therapeutic strategy of glioblastoma patients, with as few neurotoxicity as possible.

## Methods

### Patient selection

Patients with primary diagnosed glioblastoma consecutively treated in our Comprehensive Cancer Center between April 2006 and March 2010 were identified through the electronically records database. Inclusion criteria were: a histopathologically proved glioblastoma, treated by surgical total or subtotal resection (thus excluding patients with a “biopsy-only”) and full course of radiation, with immediate (<48 h) post-operative magnetic resonance image (MRI) to assess tumor resection and with documented radiological follow-up for at least 6 months after surgery. Patient data, including demographics, imaging data, treatment and clinical outcomes were retrospectively collected. The cut-off date for the analysis was September 2014. This study was approved by our Institutional Review Board and our local ethics committee. Each participant/participant’s guardian had provided consent for this study.

### Imaging data

Pre-operative imaging data (T1-weighted MRI pre- and post-contrast and FLAIR sequence) were assessed to define the tumor volume and the tumor localization with regard to the SVZ. Given the difficulty to differentiate between non-tumoral vasogenic-edema versus tumor-infiltrative area within the non-enhancing FLAIR hyperintensity lesion in T2-weighted sequences, contacting tumors were defined as tumors having a distance of 0 mm between the contrast enhancing tumor edge and the SVZ, as performed in most previous studies [26–30].

Post-operative post-contrast T1-weighted imaging was used to distinguish : patients with gross total resection (GTR) in case of no residual enhancement or near total resection (NTR) in case of only rim enhancement of the resection cavity, from patients with subtotal resection (STR), namely with residual nodular enhancement [44]. Despite a new RANO classification for evaluation of completeness of resection has been proposed recently which accounts for both enhancing and non-enhancing components of tumor [45], the post-contrast T1-wheighted imaging based classification has been used because it is still widely utilized in studies [26, 30, 46].

### Treatment

All patients received a non-biopsy surgical resection, followed by a standard of care adjuvant temozolomide-based chemoradiation, as previously described [1]. Patients were CT-scanned in treatment position with a slice distance of 2.5 mm. Radiotherapy was delivered by a three dimensional conformal radiotherapy, with a standard dose of 60 Gy in 30 daily fractions, 5 days a week. The gross tumor volume (GTV) was the contrast-enhancing lesion on T1-weighted MRI or the surgical cavity with residual contrast-enhancement. The clinical target volume (CTV) was built with a 20-mm margin around the GTV, and then was edited to include the FLAIR signal abnormality and adjust it to anatomic barriers. The planning target volume (PTV) was finally built with a 3-mm isotropic margin expansion.

Multimodal MRI was performed 1 month following the completion of chemoradiotherapy, and every 3 months thereafter until relapse.

### SVZ delineation and dosimetry data collection

**Fig. 1:**
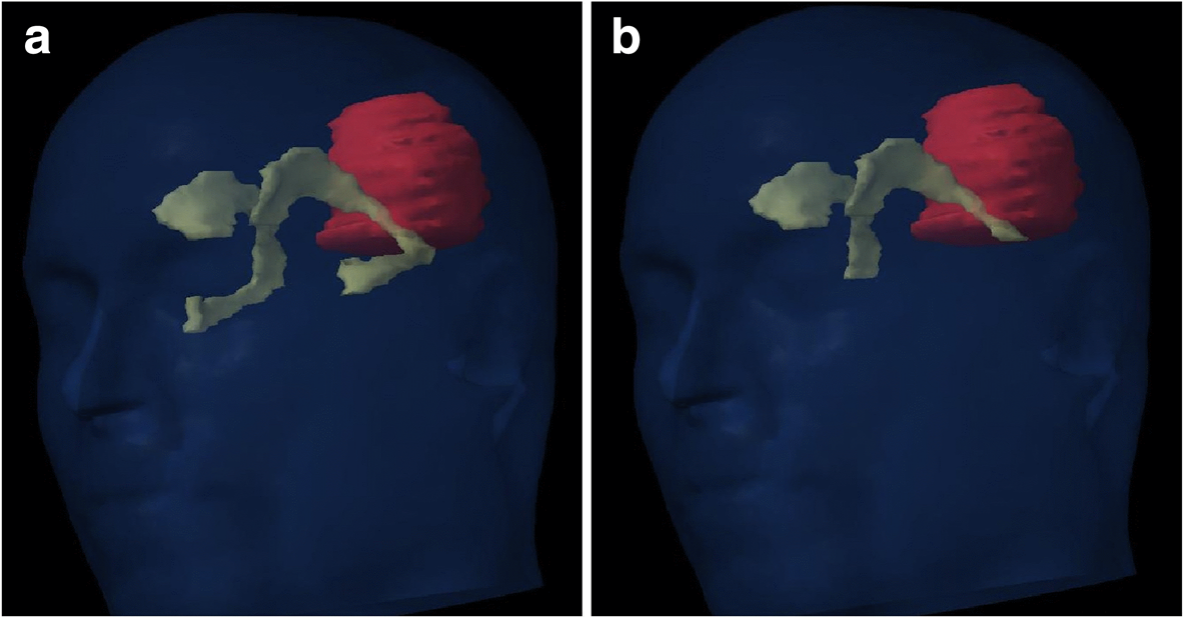
Planning computed tomography scan with three dimensional reconstruction illustrating both delineation methods for subventricular zone (SVZ). Ipsilateral and contralteral SVZ were contoured as 5 mm expansion along the lateral margins of lateral ventricles. Two delineation methods were used : TH+ method including temporal horns (**a**), and TH- method excluding them (**b**). *Yellow*: bilateral SVZ; *Red* : Clinical Target Volume

According to Barani’s delineation suggestions [47], ipsilateral (iSVZ), contralateral (cSVZ), and bilateral (bSVZ) SVZs were retrospectively segmented by a single physician as a 5-mm lateral margin of the lateral ventricles based on the patient’s original treatment planning CT scan. In a neural stem cells sparing attempt, two delineation methods were assessed: with (TH+) [34, 36, 38, 39] and without (TH-) [37] temporal horn (Fig. 1). Dose-volume histograms were retrospectively generated on the original plans, and dose-volume parameters to each SVZ volume were extracted: mean dose and volume of SVZ (%) receiving more than x Gy (VxGy).

### Patterns of recurrence

Progression was defined according to the Response Assessment in Neuro-Oncology Working Group (RANO) criteria [48] and all recurrences were centrally reviewed by an experienced neuroradiologist. For pattern of recurrence analysis, recurrence characterization was defined as: local (recurrence epicenter within 2 cm from the initial edge of T1-gadolinium contrast enhancement lesion) or distant recurrence; within or outside the SVZ.

### Statistical analysis

The data were summarized through frequency and percentage for categorical variables and by median and range for continuous variables. Comparisons between groups were performed using the chi-square test or the Fisher’s exact test for qualitative data, and the Wilcoxon test for paired quantitative data.

All survival times were calculated from the date of surgery and were estimated by the Kaplan Meier methods with 95% confidence intervals (CI), using the following first-event definitions: progression according to RANO for time to progression (TTP) and death from any cause for overall survival (OS). Patients that were still progression-free for TTP or alive for OS were censored at the time of their last follow-up. Univariate analysis was used to identify prognostic factors for TTP and OS: the log-rank test was used for categorical variables and the Cox proportional hazards model for continuous variables. For SVZ dosimetric data analysis, several dose-volume parameters were used as variables. Patients were also dichotomized using published mean dose to SVZ volumes cut-off of 40 Gy [38] and 43 Gy [34], as well as using optimal cut-off searched with the minimal p-value approach (1000 bootstrap internal validation). Multivariate analysis was assessed using the Cox proportional hazards model including significant covariates in univariate analysis.

All reported *p*-values were two-sided. For all statistical tests, differences were considered significant at the 5% level.

All statistical analyses were conducted using STATA 13.0 software.

## Results

### Patient characteristics

Forty-three patients were consecutively included. Median age at surgery was 59 years (range: 25–85). Twenty six patients (63.4%) presented a tumor contacting the SVZ.

All patients completed radiation course, with interruption of temozolomide for only 5 patients (11.36%), due to toxicity. Patient characteristics are listed in Table 1.

**Table 1:** Patient demographics, disease and treatment characteristics

Characteristic	*n* = 43	V20Gy to bSVZ ≤84% (*n* = 32)	V20Gy to bSVZ >84% (*n* = 8)	*p*
Age at surgery (years) (median, range)	59 (25–85)	60 (30–85)	64.5 (45–76)	0.710
Gender				0.222
Male	28 (65.1%)	19 (59.4%)	7 (87.5%)	
Female	15 (34.9%)	13 (40.6%)	1 (12.5%)	
ECOG performance status at diagnosis				0.080
0–1	33 (76.7%)	28 (87.5%)	3 (37.5%)	
2–4	10 (23.3%)	4 (12.5%)	5 (62.5%)	
Tumor side				0.406
Right	29 (67.4%)	22 (68.8%)	7 (87.5%)	
Left	13 (30.2%)	10 (31.3%)	1 (12.5%)	
Both	1 (2.4%)	0	0	
Tumor location				0.014
Frontal	12 (27.9%)	5 (15.6%)	6 (75%)	
Temporal	18 (41.9%)	16 (50%)	1 (12.5%)	
Parietal	10 (23.3%)	9 (28.1%)	1 (12.5%)	
Occipital	2 (4.7%)	2 (6.3%)	0	
Thalamic	1 (2.3%)	0	0	
Multilobar lesion				0.689
No	29 (67.4%)	20 (62.5%)	6 (75%)	
Yes	14 (32.6%)	12 (37.5%)	2 (25%)	
Tumor SVZ contact				0.424
Yes	26 (63.4%)	21 (67.7%)	4 (50%)	
No	15 (36.6%)	10 (32.3%)	4 (50%)	
Missing	2	1	0	
Minimal distance to SVZ (mm)				0.366
Median (range)	0 (0–27.8)	0 (0–27.8)	5.3 (0–24.6)	
Missing	2	1	0	
T1 post-gadolinium volume (cm^3^)				0.585
Median (range)	40.8 (4.4–151.9)	40.5 (4.4–94.5)	46.6 (6.5–151.9)	
Missing	3	1		
FLAIR volume (cm^3^)				0.290
Median (range)	151.4 (24.8–342.6)	115.3 (24.8–269)	194.1 (69.8–342.6)	
MGMT status				0.413
Methylated	14 (35%)	9 (31%)	4 (50%)	
Unmethylated	26 (65%)	20 (69%)	4 (50%)	
Unknown	3	3	0	
Extent of resection				0.430
GTR/NTR	19 (44.2%)	13 (40.6%)	5 (62.5%)	
STR	24 (55.8%)	19 (59.4%)	3 (37.5%)	
Number of cycles of adjuvant temozolomide (median, range)	6 (1–15)	3 (1–15)	5 (2–9)	0.510

*Abbreviations*: *MGMT* O6-methylguanine-DNA-methyltransferase, *GTR* Gross Total Resection, *NTR* Near Total Resection, *STR* SubTotal Resection, *bSVZ* bilateral SVZ, *VxGy* volume of SVZ (%) receiving more than x Gy

Median iSVZ, cSVZ and bSVZ volumes/doses are summarized in Table 2.

**Table 2:** SVZ related characteristics according to the method of delineation

	SVZ without temporal horn (TH-) (*n* = 40)	SVZ with temporal horn (TH+) (*n* = 40)	*p*
Volume (cm^3^)			
iSVZ	5 (3.4–11)	7.8 (4.9–15.5)	<0.001
cSVZ	5.5 (3.4–9.6)	7.8 (5.6–15.1)	<0.001
bSVZ	10.6 (6.8–20.6)	15 (11.8–30.6)	<0.001
Mean dose (Gy)			
iSVZ	51.3 (17.9–61.4)	45.7 (22.9–61.5)	0.298
cSVZ	15.4 (1.4–48.7)	13.7 (1.3–41.5)	<0.001
bSVZ	35 (10.8–51.8)	32.9 (14–46.4)	0.830
V10Gy (%)			
iSVZ	100 (41.3–100)	100 (55.4–100)	0.637
cSVZ	83.4 (0–100)	73.6 (0–100)	0.074
bSVZ	90.9 (26.6–100)	86.5 (36.5–100)	0.264
V20 Gy (%)			
iSVZ	96.8 (16.9–100)	83.7 (29.8–100)	0.256
cSVZ	11.6 (0–100)	8.2 (0–92.1)	<0.001
bSVZ	52.6 (8.4–100)	52.3 (15–96.1)	0.444
V30 Gy (%)			
iSVZ	87.6 (11.2–100)	72.1 (20.8–100)	0.392
cSVZ	0 (0–83.8)	0 (0–66.6)	<0.001
bSVZ	48.5 (5.6–91.3)	46.3 (10.4–76.5)	0.861
V40 Gy (%)			
iSVZ	79.7 (8.5–100)	67 (17.5–100)	0.357
cSVZ	0 (0–75.6)	0 (0–62.2)	<0.001
bSVZ	43.5 (4.2–83.8)	40.8 (8.8–72.7)	0.883
V50 Gy (%)			
iSVZ	69.1 (5.5–100)	63.8 (14.2–100)	0.451
cSVZ	0 (0–71.4)	0 (0–56.4)	<0.001
bSVZ	37 (2.7–74.3)	34.9 (7.1–66.3)	0.798
V60 Gy (%)			
iSVZ	28.7 (0–99.1)	28.2 (0–99.7)	0.687
cSVZ	(0–53.9)	0 (0–41.8)	0.317
bSVZ	13.8 (0–60.6)	14.2 (0–49.9)	0.677

*Abbreviations*: *iSVZ* ipsilateral SVZ, *cSVZ* contralateral SVZ, *bSVZ* bilateral SVZ, *VxGy* volume of SVZ (%) receiving more than x Gy

### Univariate analysis

Median follow-up, OS and TTP were 22.7 months (range 7.5–69.7 months), 22.7 months (95% CI 14.5–26.2 months) and 6.4 months (95% CI 4.4–9.3 months), respectively. At the time of cut-off, 41 patients were dead (95.3%). No patient was lost to follow-up.

On univariate analysis, age was not a prognostic factor for OS or TTP, as well as sex, ECOG performance status or O6-methylguanine-DNA-methyltransferase (MGMT) methylation status. By contrast, initial contact to SVZ was a poor prognostic factor for OS (18.7 vs 41.7 months, *p* = 0.014) and TTP (4.6 vs 12.9 months, *p* = 0.002) (Table 3).

**Table 3:** Univariate analysis of prognostic factors (except for SVZ-dosimetric data) for time-to-progression (TTP) and overall survival (OS) in patients with newly diagnosed glioblastoma

Prognostic factors	Median TTP (months)	*p* value	Median OS (months)	*p* value
Age				
< 60 years	5.2	0.651	24.7	0.914
≥ 60 years	6.4		14.4	
Gender				
Male	4.8	0.406	20.5	0.603
Female	9.1		26.2	
ECOG performans status				
0–1	5.3	0.366	24.7	0.465
2–4	6.7		14.6	
Multilobar lesion				
No	5.6	0.826	20.7	0.896
Yes	6.4		22.7	
Tumor SVZ contact				
Yes	4.6	*0.002*	18.7	*0.014*
No	12.9		41.7	
MGMT status				
Methylated	5.3	0.245	24.7	0.281
Unmethylated	6.4		20.5	
Extent of resection				
GTR/NTR	5.6	0.669	25.1	0.598
STR	6.4		18.7	

*Abbreviations*: *MGMT* O6-methylguanine-DNA-methyltransferase, *GTR* Gross Total Resection, *NTR* Near Total Resection, *STR* SubTotal Resection

#### Surgical parameters

The extent of resection (GTR/NTR vs. STR) was not found to be a prognostic factor for either OS or TTP (Table 3).

#### SVZ dosimetric parameters

Using TH+ method, no dose-volume parameter as a continuous variable was found to be a prognostic factor for either OS or TTP. With TH- method, no dose-volume parameter was found to be a predictive factor for OS, whereas V20Gy to cSVZ (HR = 0.99 (95% CI 0.98–1), *p* = 0.083) and V20Gy to bSVZ (HR = 0.99 (95% CI 0.98–1), *p* = 0.099) trended as prognostic factors for TTP.

Additionally, the patient cohort was divided into high and low radiation dose groups based on two published cut-off values for mean dose to SVZ volumes (40 Gy and 43 Gy) (Table 4). With TH+ method, patients receiving mean dose to iSVZ >43 Gy had a poorer TTP (4.8 vs 12.1 months, *p* = 0.072) and a poorer OS (20.5 vs 26.2 months, *p* = 0.087), but not significantly.

**Table 4:** Univariate analysis of SVZ-dosimetric data prognostic factors (low dose vs high dose) for time-to-progression (TTP) and overall survival (OS) using various thresholds

	Without temporal horn (TH-)					With temporal horn (TH+)				
	*n* =	Med TTP (mo)	*p*	Med OS (mo)	*p*	*n* =	Med TTP (mo)	*p*	Med OS (mo)	*p*
Dmean (40 Gy)										
iSVZ										
≤ 40 Gy	15	5.2	0.283	18.9	0.549	10	5.2	0.727	20.3	0.659
> 40 Gy	25	6.6		24.7		30	5.6		24.7	
bSVZ										
≤ 40 Gy	30	4.6	*0.023*	20.7	0.198	34	5.3	0.399	24.7	0.963
> 40 Gy	10	9.4		22.7		6	9.1		11.7	
Dmean (43 Gy)										
iSVZ										
≤ 43 Gy	17	5.3	0.529	20.3	0.392	14	12.1	0.072	26.2	0.087
> 43 Gy	23	6.4		25.1		26	4.8		20.5	
bSVZ										
≤ 43 Gy	33	5.3	0.073	22.7	0.397	-	-	-	-	-
> 43 Gy	7	9.1		34.2		-	-		-	
V20 Gy										
iSVZ										
≤ 84%	15	5.3	0.528	20.7	0.709	20	-	-	-	-
> 84%	25	6.4		24.7		20	-		-	
bSVZ										
≤ 84%	32	5.2	*0.017*	22.7	0.19	39	-	-	-	-
> 84%	8	17.7		20.3		1	-		-	

*Abbreviations*: *iSVZ* ipsilateral SVZ, *bSVZ* bilateral SVZ, *VxGy* volume of SVZ (%) receiving more than x Gy

With TH- method, patients receiving mean dose to bSVZ >40 Gy had a significantly improved TTP of 9.4 months compared to 4.6 months in patients who received less than 40 Gy (*p* = 0.023).

The optimal cut-off identified using the minimal *p*-value approach was 84% (95% CI 25–87%) for V20Gy to bSVZ with TH- method. Patients with V20Gy >84% had a better TTP (17.7 months vs 5.2 months, *p* = 0.017) (Table 4).

### Multivariate analysis

**Fig. 2:**
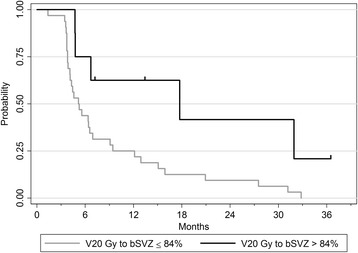
Kaplan-Meier survival curve for time to progression (TTP) by bilateral subventricular zone (bSVZ) dose, with TH- delineation method (excluding temporal horns): V20 Gy to bSVZ > 84% (*n* = 8 patients) versus ≤ 84% (*n* = 32 patients). Patients with V20 Gy to bSVZ greater than 84% had a statistically significant improvement in TTP (17.7 months vs 5.2 months)

On multivariate analysis, initial contact to SVZ remained a poor prognostic factor for TTP (HR = 3.07, 95% CI 1.27–7.39, *p* = 0.012), as well as V20Gy to bSVZ ≤84% (HR = 2.67, 95% CI 1.01–7.03, *p* = 0.047) (Table 5 & Fig. 2).

**Table 5:** Multivariate Cox regression analysis for associations between bilateral SVZ dose and time to progression (TTP)

	HR	95% CI	*p* value
V20 Gy to bSVZ			
> 84%	1.00		
≤ 84%	2.67	[1.01; 7.03]	*0.047*
Tumor SVZ contact			
No	1.00		
Yes	3.07	[1.27; 7.39]	*0.012*

*Abbreviations*: *bSVZ* bilateral SVZ, *VxGy* volume of SVZ (%) receiving more than x Gy

### Patterns of recurrence

Of the 43 patients, 41 patients recurred (95.3%): 35 patients presented an isolated local recurrence (81.4%), 2 patients presented an isolated distant recurrence (4.7%) and 4 patients presented both local and distant recurrence (9.3%); 28 patients (65.1%) recurred within SVZ whereas 13 patients (30.2%) recurred outside SVZ. Among patients with distant recurrence, 3/6 patients (50%) presented a contacting tumor at diagnosis, and 5/6 patients (83.3%) recurred within SVZ.

We then studied the pattern of recurrence of tumors according to their initial contact to SVZ, and found that 72% (18/25) of patients with initial SVZ contacting tumor recurred within SVZ, while only 57% (8/14) of patients with non-contacting SVZ tumors recurred within SVZ.

Finally, looking at the pattern of recurrence according to the radiation dose delivered to the SVZ, we found that patients with V20Gy to bSVZ ≤84% preferentially recurred within the SVZ (72.4% in case of exclusive local recurrence (21/29), 71.9% in case of local recurrence with or without distant recurrence (23/32), and 71.9% in case of local and/or distant recurrence (23/32)), while on the contrary, patients with V20Gy to bSVZ >84% preferentially recurred outside the SVZ (100% in case of exclusive local recurrence (3/3), 75% in case of local recurrence with or without distant recurrence (3/4), and 50% in case of local and/or distant recurrence).

## Discussion

Owing to complex relationships between brain tumor stem cells and neural stem cells, the optimal management of neurogenic regions of patients with glioblastoma still remains controversial. Herein, we focused on the survival rates and the recurrence patterns of glioblastoma with respect to SVZ-related factors.

In spite of inherent biases due to its retrospective design, this study has several strengths. Indeed, it included a uniform cohort of consecutively-treated glioblastoma patients with the same first-line therapy (surgical resection and adjuvant temozolomide-based chemoradiation with uniform dose/fractionation scheme), and uniform follow-up using most updated criteria, of more than 6 months after surgery, to avoid any pseudo-progression. Volume delineation, plan evaluation and approval were ensured by a single experienced radiation oncologist (ECJM). MGMT methylation status was available for almost the whole population, as well as all classical glioblastoma prognostic factors. Our salvage therapies were not homogenous (of the 38 patients who benefited a salvage treatment, 17 received a re-resection, most of them received in situ carmustine wafers (CW), and the 21 remaining patients received bevacizumab as second line, temozolomide or fotemustine); however this cofounding factor could influence OS but not TTP. Finally, our multivariate analysis aimed to control any other confounding factors. It should there be mentioned that as compared to intensity modulated radiotherapy which uses inverse planning with predefined constraints to structures, 3DCRT uses a number of fields and a beam arrangement defined by the operator, which can introduce some confounding factors in the assessment of dose to SVZ too difficult to take into account and therefore not included in our multivariable model.

Our results suggest first that initial contact to SVZ is a strong prognostic factor for TTP in multivariate analysis. This result is in accordance with several other similar reports [27, 29–31], and could be explained because glioblastoma arising in SVZ might have a higher percentage of more potent cells, making them more invasive and infiltrative. This hypothesis is reinforced by an established correlation between SVZ involvement and invasive tumor phenotype with distant or multifocal progression [26–29].

From a biological point of view, such an invasive phenotype is supported by experiments showing that SVZ neuronal progenitor cells have a higher migratory potential compared to non-SVZ ones [49].

Contrary to several studies [30, 44, 50], we failed to correlate the surgical status to survival. This could be explained either by an underpowered sample size, or because initial contact to SVZ, even in case of GTR or NTR, would lead to a microscopic residual disease due to the aggressive phenotype of tumor cells from SVZ.

Interestingly, 83.3% of our patients with distant relapse presented a recurrence within SVZ, and all but one had a GTR, highlighting the role of neurogenic regions on initiation, promotion but also repopulation of tumors. Brain stem tumor cells in neurogenic regions seem to constitute a source for a subsequent relapse.

Overall, survival data and recurrence patterns from both literature and our study suggest that a “prophylactic” irradiation to SVZ could be necessary to perform a therapeutic intensification among contacting tumor patients to encompass therapeutic resistance, but also to eradicate potential “niches” of relapse among other glioblastoma patients. Several studies have established a correlation between a dose level to SVZ and survival outcomes [34–40]. The first ever study from Evers et al. (UCLA, USA) found that mean dose >43 Gy to bSVZ was an independent factor for PFS (HR = 0.73, 95%CI = [0.57; 0.95]), but not for OS on multivariate analysis [34]. These results were not confirmed in the series from VUMC (The Netherlands) which could not find an association between this dose to SVZ and PFS [35]. This may be due to the more aggressive population of exclusively grade 4 patients in the VUMC study (compared to a mix grade 3/grade 4 glioma population in the UCLA study), which might require a higher dose to bSVZ. Given these heterogeneous results, Lee et al. updated and pooled these data restricted to only glioblastoma patients, but they failed to find a correlation between bSVZ dose and PFS as in Evers’s study. However, they found that increasing mean dose to iSVZ (>59.4 Gy) was an independent factor for PFS on multivariate analysis (HR = 0.45, 95%CI = [0.25; 0.82]) [36], similarly to Gupta et al., who found it was an independent factor for OS (HR = 0.87, 95%CI = [0.77; 0.98]) [37]. This benefit of increased iSVZ dose on PFS and/or OS was confirmed by Chen et al. with a 40 Gy cut-off, but only in the GTR group, hypothesizing that STR patients preferentially relapsed from residual tumor mass outgrowth and thus highlighting the role of such irradiation to eradicate stem cells “niches” (HR for PFS of 0.385, 95%CI = [0.165–0.901]) and HR for OS of 0.385, 95%CI = [0.165–0.895]) [38], and more recently by Adeberg et al. with the same cut-off but only in univariate analysis (median PFS : 8.5 versus 5.2 months; *p* = 0.013) [40]. Interestingly, contradictory results came from a recent study by Elicin et al. which found a worse PFS among patients with high iSVZ dose (>62.25 Gy) in both subgroups of good performance status and SVZ without tumoral contact. A worse PFS was also found among patients with dose to cSVZ >59.2 Gy (7.1 vs 10.4 months), but it was not confirmed in multivariate analysis [39]. Gupta et al. also found a worse PFS and OS with higher cSVZ doses, but patients in higher dose group had actually larger tumors, more often crossing the midline and with less GTR so that it was not confirmed in multivariate analysis [37]. On the contrary, Adeberg et al. found a better PFS among patients receiving a mean dose to cSVZ ≥30 Gy (HR in multivariate analysis of 0.45, 95%CI = [0.20–0.98]) [40]. A summary table of all previous results and our main results is presented (Table 6).

**Table 6:** Summary of previous results of SVZ irradiation and main results from the current study

Studies	*n* =	Cut-off mean dose to SVZ	TTP (months) HR (IC95%)	*p*	OS (months) HR (IC95%)	*p*
Evers 2010 [34]	55	bSVZ ≥ 43Gy vs < 43Gy	15 vs 7.2	0.028^*^	NK	NA
		bSVZ dose (continuous) ^ma^	0.73 (0.57–0.95)	0.019^*^	NK	NA
Slotman 2011 [35]	40	bSVZ ≥ 43Gy vs < 43Gy	13.2 vs 13.1	0.740	18.6 vs 20.1	0.930
Lee 2012 [36]	173	iSVZ > 59.4Gy vs ≤ 59.4Gy	12.6 vs 9.9	0.042^*^	25.8 vs 19.2	0.173
		iSVZ > 59.4Gy ^ma^	0.45 (0.25–0.82)	0.009^*^	0.65 (0.35–1.21)	0.177
Gupta 2012 [37]	40	iSVZ > 59.9Gy vs ≤ 59.9Gy	10 vs 11	0.920	17 vs 15	0.950
		iSVZ dose (continuous) ^ma^	0.91 (0.80–1.03)	0.116	0.87 (0.77–0.98)	0.025^*^
		cSVZ > 57.9Gy vs ≤ 57.9Gy	NR vs 10	0.02^*^	NR vs 14	0.05
		cSVZ > 57.9Gy ^ma^	0.96 (0.91–1.30)	0.797	0.95 (0.72–1.26)	0.736
Chen 2013 [38]	116	iSVZ ≥ 40Gy vs < 40Gy	0.82 (0.51–1.34)	0.434	0.93 (0.57–1.50)	0.754
		*GTR subgroup:*				
		*iSVZ ≥ 40Gy vs < 40Gy*	*15.1 vs 10.3*	*0.023* ^*^	*17.5 vs 15.6*	*0.027* ^*^
		*iSVZ ≥ 40Gy* ^*ma*^	*0.39 (0.17–0.90)*	*0.028* ^*^	*0.39 (0.17–0.90)*	*0.027* ^*^
Elicin 2014 [39]	60	cSVZ ≥ 59.2Gy vs < 59.2Gy	7.1 vs 10.4	0.009^*^	NK	NA
		cSVZ ≥ 59.2Gy ^ma^	1.72 (0.80–3.53)	0.161		
Adeberg 2016 [40]	54	iSVZ ≥ 40Gy vs < 40Gy	8.5 vs 5.2	0.013^*^	21.3 vs 18.0	0.19
		iSVZ ≥ 40Gy ^ma^	0.52 (0.26–1.03)	0.06		
		cSVZ ≥ 30Gy vs < 30Gy	10.1 vs 6.9	0.025^*^	21.6 vs 21.2	0.29
		cSVZ ≥ 30Gy ^ma^	0.45 (0.20–0.98)	0.04^*^		
Khalifa 2017	43	*SVZ without temporal horn:*				
		bSVZ > 40Gy vs ≤ 40Gy	9.4 vs 4.6	0.023^*^	22.7 vs 20.7	0.198
		V20Gy to bSVZ > 84% vs ≤84%	17.7 vs 5.2	0.017^*^	20.3 vs 22.7	0.19
		V20Gy to bSVZ ≤84% ^ma^	2.67 (1.01–7.03)	0.047^*^		

*Abbreviations*: *iSVZ* ipsilateral SVZ, *bSVZ* bilateral SVZ, *cSVZ* contralateral SVZ, *VxGy* volume of SVZ (%) receiving more than x Gy, *TTP* Time To progression, *HR* Hazard Ratio, *OS* Overall Survival, *NC* Not Known, *NA* Not Applicable, *ma* = multivariable analysis

*p <0.05

However, several limits should be noted in these retrospective studies, which could explain some of these discordances. Population was not always homogenous : grade 3 gliomas were included in Evers’ study [34] and patients could receive other drugs than temozolomide in two studies [34, 36]. Methylation status, which is a strong predictive factor of outcome [51], was missing or insufficiently reported except in two studies [37, 40]. Variability in the radiotherapy target volume delineation and dose-prescription between studies (one vs. two dose-level prescription depending on European or American guidelines) but also within a series [34, 36, 40], and variability of the SVZ delineation between studies (with [37] vs. without temporal horn [34, 36, 38, 39]) and within a series (3–5 mm thick), should also be mentioned. Above all, only mean dose to SVZ was considered in all studies, and thresholds were defined using only median mean doses or 75^th^ percentile.

Instead, our approach consisted of exploring dose distributions to SVZ during radiotherapy through a more complete analysis of the SVZ dose-volume histogram, in order to better understand the role of low/mid doses to SVZ in addition to high dose. This innovative approach had never been assessed before. We also tried to find an optimal cut-off for relevant dose-volume parameters, through a statistical method. It is there worth mentioning that due to the exploratory nature of the analysis, no adjustment for multiple testing was used. This led us to identify a V20Gy >84% to bSVZ (without temporal horn) as an independent prognostic factor for better TTP. The multivariate analysis included contact to SVZ and radiation dose to SVZ as co-variables and could then take into account the fact that patients with contacting tumors inherently received a higher dose to SVZ. Interestingly, in univariate analysis, a mean dose to bSVZ >40Gy was also a factor for better TTP, in the same way as Evers [34]. Last, it should be noted that a poorer TTP and OS has been found among patients with mean dose to iSVZ (with temporal horn) >43 Gy; however this did not reach statistical significance, and additionnally this was probably due to a larger proportion of contacting tumors in the iSVZ high dose group (80%) compared to the low dose group (30%).

Another feature of our approach was to explore two SVZ delineation methods (based on a standard and reproducible 5-mm lateral margin of the lateral ventricle [47]) : with (TH+) or without (TH-) temporal horn. The aim was to assess an hippocampal sparing method (TH-), as most neurogenic regions with potential brain tumor stem cells stand lateral to the body of lateral ventricles and biologically equivalent doses in 2-Gy fractions to 40% of the bilateral hippocampi greater than 7.3 Gy was shown to be associated with long-term neuro-cognitive impairment [52]. Hippocampal avoidance during whole-brain radiotherapy for brain metastases was associated with preservation of memory and quality of life in a phase II trial [43]. Hippocampal avoidance is not assessed in glioblastoma trials because optimal PTV coverage is the main objective. But when it comes to performing “prophylactic” irradiation to SVZ, neurocognitive preservation should be envisaged, as we confirmed that SVZ irradiation is compatible with hippocampal sparing through TH- delineation method. Chen et al. argued that iSVZ dose ≥40 Gy with TH+ method did not correlate with worsened patient KPS score at the end of radiation treatment, however neurocognitive performances are not precisely assessed in KPS [38]. We thus believe that delineation for glioblastoma patients should then include bSVZ delineation without temporal horn, as well as hippocampi delineation, with use of intensity modulated radiotherapy planning to ensure a sufficient coverage to bSVZ while ensuring an hippocampal avoidance according to previous constraints.

Regarding patterns of recurrence, larger proportion of patients with initial SVZ contacting tumor recurred within SVZ compared to patients with non-contacting tumors. This seems to be due to the in-field pattern of recurrence typically seen in glioblastoma following radiotherapy as most relapses occur within 2 cm beyond the contrast enhancement, and almost exclusively in the edema area [53–55]. Additionally, patients with V20Gy >84% turned out to relapse away from SVZ contrary to those with V20Gy ≤84%, who mainly relapsed within SVZ. Radiobiological considerations could provide some interesting explanations to this pattern, as there is strong evidence that *sublethal* irradiation promotes glioblastoma cells migration, via an increase in the αvβ3 integrin expression [56]. Therefore, hypothesizing that SVZ is a potential source of recurrences through repopulation of tumor by remaining brain tumor stem cells following chemoradiation [11], the level of bSVZ irradiation could explane the location of recurrence within vs without SVZ.

Overall, these data could support two hypotheses. The simplest one would be that patients who received a V20Gy >84% to bSVZ really benefited from such a prophylactic irradiation through a brain tumor stem cells eradication. An additional and/or alternative one would be that some of patients who received a higher bSVZ irradiation experienced an “artificial” increase in TTP via a more durable transition through a migratory phenotype, before engaging towards a proliferative one. Then a strategy to encompass such a mechanism of radioresistance would be to associate prophylactic irradiation of bSVZ with intensity modulated radiotherapy (and hippocampal sparing) ensuring a V20Gy >84%, and inhibitors of migration which could target integrin-mediated signalling pathway. This strategy has never been assessed but could provide promising results.

Meanwhile, some more robust data should soon come from three ongoing or recently concluded clinical trials regarding the impact of SVZ irradiation in GBM (NCT01478854 exploring a deliberate sparing of neurogenesis niches, contrary to NCT02039778 and NCT02177578, exploring a deliberate SVZ irradiation).

## Conclusion

To conclude, our data from a homogeneous patient cohort suggest that contact to SVZ could be an independent poor prognostic factor for TTP, as well as bSVZ insufficient dose coverage such as a V20Gy ≤84%. We also provide data suggesting that the pattern of recurrence is influenced by contact to SVZ and by radiation dose to bSVZ. These results highlight the prominent role of the SVZ in the prognostic of glioblastoma patients through mechanisms of therapeutic resistance, and therefore raise the question of their role in the optimal management of glioblastoma. Further prospective clinical trials assessing prophylactic irradiation of bSVZ by intensity modulated irradiation, with or without migration-inhibitors, would be critical in determining whether such strategy could improve devastating outcomes of glioblastoma patients.

## Abbreviations

bSVZ: Bilateral SVZ
cSVZ: Contralateral SVZ
GTR: Gross total resection
iSVZ: Ipsilateral SVZ
MGMT: O6-methylguanine-DNA-methyltransferase
NTR: Near total resection
OS: Overall survival
STR: Subtotal resection
SVZ: Sub-ventricular zone
TH -: SVZ delineation method without temporal horn
TH+: SVZ delineation method with temporal horn
TTP: Time to progression
VxGy: Volume of SVZ (%) receiving more than x Gy
